# Sialic Acid in Neurodegenerative and Psychiatric Disorders: From Molecular Regulation to Targeted Nanocarrier-Based Therapy

**DOI:** 10.3390/pharmaceutics17121593

**Published:** 2025-12-10

**Authors:** Natalia Treder, Tomasz Bączek

**Affiliations:** 1 Department of Pharmaceutical Chemistry, Medical University of Gdańsk, Hallera 107, 80-416 Gdańsk, Poland; tomasz.baczek@gumed.edu.pl; 2 Department of Nursing and Medical Rescue, Institute of Health Sciences, Pomeranian University in Słupsk, 76-200 Słupsk, Poland

**Keywords:** sialic acid, neurodegenerative disorders, nanocarriers, targeted drug delivery

## Abstract

In recent years, the exploration of molecular and cellular mechanisms underlying central nervous system (CNS) disorders has expanded beyond classical neurotransmitter- and receptor-based approaches toward a more integrated view including immune, metabolic, and glycosylation processes. Among these, sialic acid and its derivatives have emerged as critical regulators of neuronal communication, immune modulation, and synaptic plasticity. Their involvement ranges from maintaining neurochemical homeostasis under physiological conditions to contributing to the onset and progression of neurodegenerative and psychiatric diseases. Given the central role of sialylation in cellular recognition, receptor signaling, and blood–brain barrier (BBB) interactions, understanding these pathways provides valuable insight for the development of advanced therapeutic and diagnostic strategies. This review highlights recent evidence linking altered sialic acid metabolism and polysialylation to Alzheimer’s disease and other neurodegenerative and psychiatric disorders. It further discusses the potential of sialic acid-related mechanisms as novel molecular targets and their integration into innovative nanocarrier-based drug delivery systems designed to improve brain penetration, selectivity, and therapeutic efficacy. Finally, current challenges and future perspectives in translating sialic acid-based approaches into clinical applications are addressed.

## 1. Introduction

Neurodegenerative disorders represent one of the greatest global health challenges of the 21st century, with Alzheimer’s disease standing as the most prevalent and clinically significant form of dementia [1,2]. Alzheimer’s disease accounts for approximately 60–70% of all dementia cases, while other major forms include vascular dementia and Lewy body dementia, with less common types such as Parkinson’s disease, frontotemporal dementia, and Huntington’s disease. According to the 2024 Lancet Commission on dementia prevention, intervention, and care, the total number of dementia cases is estimated to exceed 150 million by 2050, reflecting the rapid aging of populations worldwide and the absence of effective therapies [3]. Beyond classical neurodegenerative diseases, psychiatric disorders such as schizophrenia, major depressive disorder, bipolar disorder, generalized anxiety disorder, and post-traumatic stress disorder contribute significantly to the global neurological disease burden [4,5]. The global incidence of neurodegenerative and psychiatric disorders has markedly increased over the past three decades, driven by a wide spectrum of risk factors ranging from common medical and biological determinants such as hypertension, obesity, age, sex, and genetic predisposition to social factors including education level, living conditions, and excessive digital exposure [6,7,8,9]. To fully appreciate the scale of this global challenge, it must be acknowledged that disorders of the CNS affect not only the elderly but also the broader population, regardless of age or social status. For instance, early-onset dementia, defined as occurring between the ages of 40 and 64, has nearly doubled in global prevalence since 1990, highlighting the increasing societal and economic burden that extends beyond aging populations [2]. Additionally, attention should be paid to newly emerging factors that may contribute to the development of neurodegenerative and psychiatric disorders. Although current knowledge does not allow for a precise estimation of the magnitude of this issue, existing reports already indicate that synthetic psychoactive substances may lead to persistent mental disorders [10,11]. Recent longitudinal studies have linked frequent cannabis use to an elevated risk of psychosis, depressive disorders, and cannabis-induced schizophrenia, particularly in genetically predisposed individuals [12,13]. These trends highlight the increasing complexity of factors contributing to brain dysfunction across the lifespan and emphasize the need to understand the underlying mechanisms of disease development in order to enable early prevention.

While epidemiological and clinical studies have identified numerous genetic and environmental risk factors contributing to neurodegenerative and neuropsychiatric disorders, a critical challenge remains in understanding how these diverse influences converge at the molecular and cellular levels to drive disease onset and progression. Elucidating these molecular mechanisms–linking risk exposure to neurobiological alterations and therapeutic response–is essential for developing effective diagnostic and treatment strategies. Several interconnected biochemical pathways have been implicated in these disorders, including altered neurotransmitter signaling, oxidative stress, mitochondrial dysfunction, and neuroinflammation [14,15,16]. Beyond these well-established mechanisms, recent findings have drawn increasing attention to post-translational and metabolic processes–particularly sialylation–as key modulators of neuronal communication and immune homeostasis in the brain. Sialic acids are a diverse family of acidic monosaccharides that exhibit substantial structural variability through multiple chemical modifications, which in turn shape their biological functions. Positioned within the surface-exposed region of glycoproteins and glycolipids, sialic acids influence both biochemical and biophysical interactions at the cell surface. In addition to sialylated glycoproteins, neurons are highly enriched in sialylated glycolipids, especially gangliosides, which play essential roles in membrane organization, synaptic stability, receptor clustering, and axonal growth. Gangliosides such as GM1, GD1a, and GT1b contribute to neuroprotection, modulate neurotrophic signaling, and participate in immune–neural communication through interactions with sialic acid-binding immunoglobulin-like lectin (SIGLEC) receptors. Dysregulation of ganglioside composition has been linked to impaired synaptic plasticity, neuroinflammation, and psychiatric disorders, underscoring their importance as functional components of the neuronal glycocalyx [17,18]. In addition, sialic acid-modified motifs are increasingly explored as biomarkers, therapeutic targets, and functional components in drug-delivery systems, where their affinity for specific receptors is used to enhance targeting selectivity. This nine-carbon acidic monosaccharide forms part of the surface-exposed layer of cell-surface glycoconjugates, that regulates receptor signaling and cell–cell recognition and plays a critical role in maintaining blood–brain barrier (BBB) integrity. The endothelial glycocalyx, rich in sialic acid-modified glycoproteins, forms a negatively charged, hydrated matrix that regulates molecular permeability and prevents leukocyte adhesion. In turn, disturbances in sialylation within the BBB glycocalyx increase barrier permeability and promote neuroinflammation, a characteristic feature of both neurodegenerative and psychiatric pathologies. Thus, the interplay between sialylation, neurotransmission, and neuroimmune signaling represents a key molecular interface linking metabolic regulation to brain health and disease [19].

The introduction of nanotechnology has revolutionized biomedical research by enabling precise manipulation of matter at the molecular scale, thereby bridging the gap between materials science and medicine. Nanomaterials—owing to their tunable physicochemical properties, high surface-area-to-volume ratio, and capacity for surface functionalization—offer unprecedented opportunities for targeted drug delivery, controlled release, molecular imaging, and biosensing [20,21,22]. Their small size allows efficient penetration into tissues and across biological barriers, while surface modification with ligands, antibodies, or carbohydrates enables selective interaction with disease-specific targets. Consequently, nanocarriers can be engineered to achieve site-specific accumulation, minimize systemic toxicity, and provide sustained pharmacological action. Within this framework, sialic acid-based nanostructures represent a particularly promising class of bio-functional materials. Owing to its negative charge, hydrophilicity, and innate ability to traverse the BBB, sialic acid enhances nanoparticle stability, prolongs circulation time, and facilitates brain-specific delivery. Furthermore, sialic acid can mediate active targeting through its interaction with SIGLECs and other glycan receptors overexpressed in pathological tissues. The benefits of combining nanocarriers with sialic acid are gaining increasing attention in therapy and diagnostics. For instance, gold nanoparticles (AuNPs) modified with N-acetylneuraminic acid (Neu5Ac)—one of the principal forms of sialic acid and the only type endogenously expressed in humans—were shown to bypass the reticuloendothelial system by suppressing innate immune recognition through SIGLEC-mediated interactions, thereby reducing phagocytic clearance and enhancing tumor accumulation *in vivo* [23]. Similarly, in another study, sialic-acid-modified paclitaxel liposomes target overactivated neutrophils via L-selectin, reducing tumor and inflammation site infiltration and improving survival in preclinical sepsis and melanoma models [24]. 

The dual functionality of sialic acid-modified nanocarriers—improved pharmacokinetics and receptor-mediated recognition—highlights their broad therapeutic relevance, not only in oncology but also in neurological applications. In this review, we aim to provide a comprehensive overview of current strategies for the treatment of neurodegenerative and neuropsychiatric disorders, with a particular focus on the role of sialic acid and polysialic acid (polySia) in maintaining neural homeostasis and modulating disease-related pathologies. Following this introduction, the review will first summarize current therapeutic approaches for these conditions. This will be followed by an analysis of recent reports on sialylation-related mechanisms, including the functions of sialic acid and polySia in neuronal and glial physiology, immune regulation, and synaptic plasticity. Subsequently, we will discuss advanced therapeutic strategies based on sialic acid-functionalized nanocarriers and glycoconjugate systems, highlighting their potential for targeted drug delivery, enhanced BBB penetration, and receptor-mediated specificity. Finally, the review will address current limitations and outline future perspectives in the field.

## 2. Current Approaches to the Treatment of Neurodegenerative Diseases and Psychiatry Disorders

Although the precise etiology of Alzheimer’s disease remains unresolved, current knowledge supports the existence of several well-characterized pathological features. One of the earliest is the progressive loss of cholinergic neurons in the basal forebrain, leading to reduced acetylcholine levels in the cortex and hippocampus—regions critical for memory and cognition [25,26]. To compensate for this neurotransmitter deficit, acetylcholinesterase inhibitors such as donepezil, rivastigmine, and galantamine are commonly prescribed [27]. These drugs block the enzyme responsible for acetylcholine degradation, temporarily increasing its concentration in synaptic clefts and partially restoring cholinergic transmission. Their efficacy, however, is generally limited to mild or moderate stages of the disease, and they do not prevent neuronal loss. Among traditionally used drugs, memantine—an NMDA receptor antagonist—is also administered in more advanced stages to reduce glutamate-induced excitotoxicity and stabilize cognitive decline. Memantine can be used alone or in combination with acetylcholinesterase inhibitors, although clinical benefits are modest [28]. However, cholinergic deficits are not the only pathological features of Alzheimer’s disease. Specifically, extracellular aggregation of amyloid-β into plaques and intracellular hyperphosphorylation of tau protein forming neurofibrillary tangles are the most thoroughly studied molecular features of the disease [29]. These proteinopathies contribute to synaptic dysfunction, neuroinflammation, and neuronal loss, which may further exacerbate cholinergic deficits, although the precise causal relationships remain incompletely understood. Given their central role in disease progression, these proteinopathies have become the primary targets of novel therapies. In recent years, monoclonal antibodies against amyloid-β—including aducanumab, lecanemab, and donanemab—have demonstrated the capacity to reduce amyloid plaque burden and modestly slow cognitive decline in early-stage Alzheimer’s disease [30,31,32]. Their clinical application, however, faces substantial challenges: efficacy appears largely restricted to very early stages, treatment requires confirmation of amyloid pathology via PET or CSF biomarkers, regimens are resource-intensive and costly, and safety concerns such as amyloid-related imaging abnormalities necessitate careful monitoring. The long-term effects on tau pathology and sustained cognitive benefit beyond 18–24 months remain uncertain [33]. In other neurodegenerative disorders, clinical management also relies primarily on pharmacological strategies aimed at symptom control. In Parkinson’s disease, the main pathological feature is the progressive degeneration of dopaminergic neurons in the substantia nigra, leading to dopamine depletion in the striatum and resulting in motor dysfunction such as tremor, rigidity, and bradykinesia [30,34]. Consequently, levodopa—often combined with carbidopa—remains the mainstay of therapy, with adjunctive agents such as dopamine agonists, MAO-B inhibitors, or COMT inhibitors used to optimize motor function and reduce fluctuations [35,36,37]. In Huntington’s disease, treatment is generally limited to tetrabenazine or deutetrabenazine for chorea management [38]. Deep brain stimulation (DBS) and continuous infusion therapies provide complementary interventions to pharmacological approaches, improving quality of life in selected patients. Nonetheless, these treatments often fail to modify the underlying neurodegenerative processes, and invasive or costly procedures like DBS present additional limitations [39]. A similar symptom-targeted approach characterizes pharmacotherapy in psychiatric disorders, despite considerable variability in patient response and frequent adverse effects. Antipsychotics, both typical and atypical, remain the primary agents for schizophrenia and related psychoses, acting on dopaminergic and serotonergic systems to alleviate positive symptoms, although they have limited efficacy on cognitive deficits or negative symptoms. Mood stabilizers, including lithium, valproate, and carbamazepine, together with atypical antipsychotics, form the cornerstone of bipolar disorder management, while antidepressants—including SSRIs, SNRIs, tricyclics, and MAO inhibitors—constitute the mainstay therapy for major depressive disorder [40,41,42]. Treatment-resistant cases are common, and long-term use can induce metabolic disturbances, extrapyramidal effects, sedation, or withdrawal syndromes. Adjunctive neuromodulatory strategies such as electroconvulsive therapy, repetitive transcranial magnetic stimulation, and DBS offer additional options in severe or refractory cases, although they are resource-intensive and variably effective [43,44]. 

Taken together, the pharmacological landscape across these conditions is dominated by treatments aimed at managing clinical symptoms, which insufficiently address the underlying molecular and cellular mechanisms. While ongoing research seeks to overcome the limitations and adverse effects of existing drugs—including the development of novel formulations and improved delivery systems—current therapies remain primarily focused on managing symptoms rather than modifying disease progression [27]. These limitations, together with the complex, multifactorial pathophysiology of central nervous system disorders, highlight the urgent need for innovative strategies that integrate molecular targeting, enhanced BBB delivery, and neuroprotective modulation.

## 3. Sialic Acid and Polysialylation as Diagnostic Markers in Psychiatric and Neurodevelopmental Disorders

Despite the rapidly growing prevalence of CNS disorders, effective disease-modifying treatments remain limited. Ongoing efforts increasingly concentrate on elucidating the cellular and molecular mechanisms driving neurodegeneration and psychiatric conditions–including neuroinflammation, oxidative stress, abnormal protein aggregation, and impaired neuronal signaling—with the aim of identifying new diagnostic markers and therapeutic targets. Although these insights have not yet yielded clinically established interventions comparable to acetylcholinesterase inhibitors in Alzheimer’s disease or SSRIs in depression, progress in this field continues to refine our understanding of early pathological changes. In this context, the potential role of sialic acid in both the onset and progression of neurodegenerative diseases warrants particular attention, as it may represent a key molecular link between dysregulated cellular processes and impaired neuronal function. To date, research on the multifaceted roles of sialic acid and polySia in neurodegenerative and psychiatric disorders has primarily focused on the most prevalent condition, Alzheimer’s disease. Nevertheless, albeit to a lesser extent, their key roles are also being recognized and investigated in Parkinson’s disease and certain psychiatric disorders, including schizophrenia, bipolar disorder, and autism spectrum disorder (ASD).

### 3.1. Alzheimer’s Disease

Alzheimer’s disease is increasingly recognized as a disorder in which immune dysregulation contributes to driving neurodegeneration. Microglia, as the brain’s resident immune cells, play a crucial role in responding to amyloid β (Aβ) plaques and other neuropathological features of Alzheimer’s disease [45]. Their activation and regulation are tightly controlled by signaling pathways containing immunoreceptor tyrosine-based activating and inhibitory motifs, which determine the balance between pro- and anti-inflammatory responses. These signaling motifs are central to the function of sialic acid-binding immunoglobulin-like lectins (SIGLECs), a family of receptors that fine-tune microglial activity in response to sialic acid-modified glycoconjugates. Recent evidence has shown increased expression of SIGLEC10 and decreased expression of crystallin mu (CRYM) in the blood of Alzheimer’s disease patients, with both genes showing potential as diagnostic biomarkers [46]. Similarly, SIGLEC11, a human-specific microglial receptor that recognizes α2-8-linked polysialylated ligands, exhibits anti-inflammatory properties. Transgenic mice expressing human SIGLEC11 display reduced microglial activation, decreased oxidative stress, and attenuated neuronal loss with aging, suggesting that SIGLEC11-mediated recognition of sialic acid-modified structures may protect against neuroinflammatory damage [47]. Several studies have also indicated that among the SIGLEC receptors, SIGLEC3 (CD33) may represent a genetic risk factor for Alzheimer’s disease [48,49,50]. The SIGLEC3 gene, which encodes a transmembrane sialic acid-binding protein expressed on microglia and myeloid cells, produces isoforms that differentially modulate microglial phagocytosis of Aβ aggregates. The longer isoform, SIGLEC3M, containing a functional sialic acid-binding domain, inhibits phagocytosis and enhances cell adhesion, whereas the shorter isoform, SIGLEC3m, which lacks this domain, promotes phagocytosis and proliferation [49]. Based on these findings, structural modeling of the SIGLEC3 R69G variant revealed altered sialic acid binding, potentially increasing receptor affinity and inhibitory signaling, thereby further reducing Aβ clearance over time [50]. Consequently, SIGLEC3 exerts its inhibitory effects through recruitment of SHP-1 phosphatase via its ITIM, leading to sustained microglial inactivation and impaired amyloid plaque clearance [48]. Similarly, another study demonstrated that the Alzheimer’s disease-associated SIGLEC3 risk variant increases the expression of the full-length sialic acid-binding isoform, which enhances inhibitory signaling and reduces microglial Aβ clearance. SIGLEC3 directly interacts with CD45, a phosphatase whose activity is suppressed through sialic acid-dependent binding. This interaction was found to be elevated in Alzheimer’s disease patients and correlated with greater pathological burden, thereby establishing a functional SIGLEC3-CD45 sialoglycan axis implicated in Alzheimer’s disease pathogenesis. Collectively, these findings indicate that altered SIGLEC signaling contributes to immune dysregulation and impaired debris clearance, linking sialic acid recognition to neurodegenerative progression [51]. 

**Figure 1:**
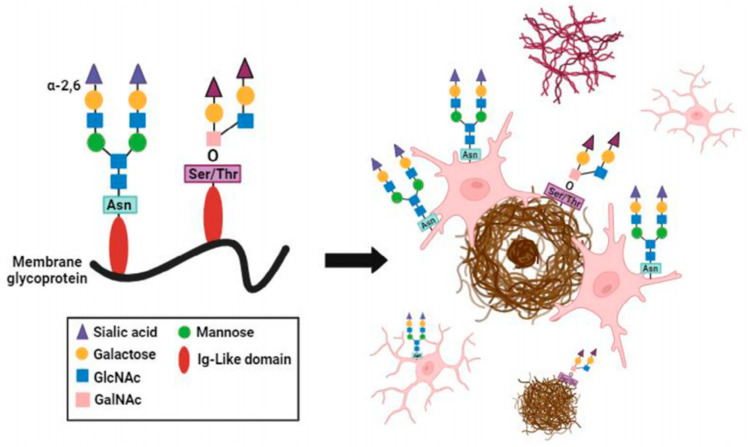
Cellular and aggregate specific representation of sialylation landscape in Alzheimer’s disease. Graphical summation of sialylation localization relative to plaque pathology and tau pathology [52].

Altered sialylation extends beyond receptor-mediated effects to encompass global changes in glycosylation across brain regions. Studies comparing O- and N-linked sialylation patterns in post-mortem Alzheimer’s disease brains have demonstrated significant increases in N-sialylation within the Aβ plaque microenvironment, particularly in plaque-associated microglia [52] (Figure 1). Interestingly, while phosphorylated tau pathology slightly increased N-sialylation, it had no significant influence on O-sialylation. These results indicate that specific glycosylation patterns, especially N-sialylation, might reflect microglial responses to local pathology and could serve as histological indicators of Alzheimer’s disease progression. In addition to these global glycosylation changes, phosphorylated tau (p-tau)-containing structures such as neurofibrillary tangles (NFTs) and granulovacuolar degenerations (GVDs) have been shown to be hypersialylated in Alzheimer’s disease hippocamp, while amyloid plaque cores remain unsialylated. The use of anti-sialic acid antibodies proposed as a diagnostic approach to identify these sialylated tau lesions, further emphasizing this close relationship between sialylation and tau pathology [53]. 

Another important observation concerns the regional specificity of sialic acid metabolism. Distinct sialylation patterns were observed between the temporal lobe—highly susceptible to Alzheimer’s disease pathology—and the cerebellum, a relatively spared region. Alzheimer’s disease brains exhibited elevated levels of free sialic acid in the temporal lobe, alongside reductions in Neuraminidase 2 and 4, enzymes responsible for sialic acid turnover. In contrast, the cerebellum displayed increased total and bound sialic acid, suggesting compensatory protective mechanisms. This regional variation supports the hypothesis that disrupted sialylation dynamics contribute to selective susceptibility in Alzheimer’s disease. Complementary evidence from glycoproteomic analyses across different brain regions of Alzheimer’s disease patients further demonstrated widespread differences in sialylation and branching of N-glycans, indicating regional heterogeneity in sialoglycan expression and a strong association between altered glycosylation patterns and disease progression [54,55]. At the molecular level, disruptions in the sialic acid biosynthetic pathway can also trigger protein misfolding and endoplasmic reticulum (ER) stress, mechanisms that are shared between UDP-N-acetylglucosamine 2-epimerase/N-acetylmannosamine kinase (GNE) myopathy and neurodegenerative disorders [56]. Mutations in the GNE gene—key for sialic acid synthesis—induce unfolded protein response activation, apoptosis, and cellular dysfunction. Another study highlighted the functional impact of microglial keratan sulfate (KS), which is also sialylated. Increased levels of sialylated KS and its synthetic enzyme GlcNAc6ST1 were observed in Alzheimer’s disease brains and transgenic mouse models, while genetic deletion of GlcNAc6ST1 resulted in enhanced Aβ clearance and reduced plaque burden, indicating that microglial sialylated KS contributes to Alzheimer’s disease pathology by suppressing microglial phagocytic activity [57]. 

**Figure 2:**
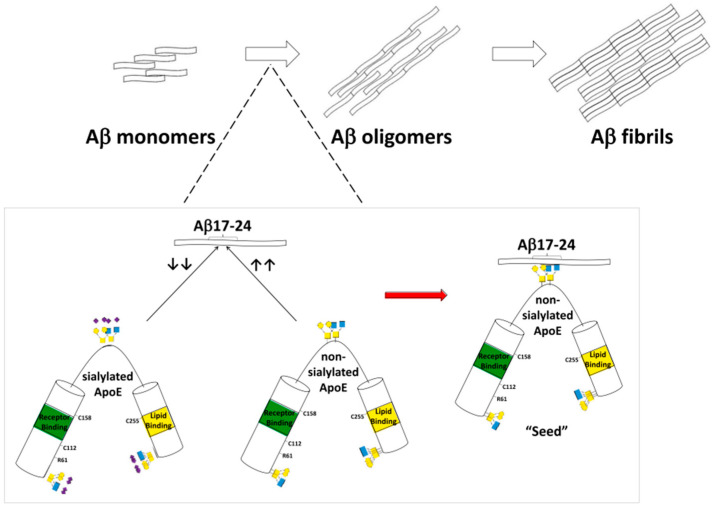
Data presented in this figure indicate that the sialic acid modification in human ApoE protein may serve as a key modulator of Aβ fibrillization. The removal of sialic acid increases ApoE binding affinity for Aβ17–24 residues and such an interaction may promote Aβ fibrillation. The presence of sialic acid attenuates ApoE binding with Aβ17–24 and reduces Aβ fibrillation [60].

The interplay between sialic acid metabolism and oxidative stress was also explored. Elevated plasma sialic acid levels in Alzheimer’s disease patients correlated with increased oxidative stress markers and reduced antioxidant capacity, suggesting that excessive sialylation may accompany or exacerbate redox imbalance [58]. Similarly, reduced serum sialic acid levels were linked to impaired antioxidative defenses and higher oxidative damage, alongside altered levels of fibroblast growth factor 23 (FGF23) and albumin, supporting the notion that both hypo- and hypersialylation may reflect different stages or systemic responses in Alzheimer’s disease pathogenesis [59]. Genetic and posttranslational aspects of sialylation were also clarified. The apolipoprotein E (ApoE) isoforms, central to Alzheimer’s disease susceptibility, exhibit distinct sialylation profiles: ApoE2 carries the highest sialic acid content, while ApoE4 contains the least. Desialylation of ApoE2 increases its affinity for Aβ and promotes fibrillation, providing a mechanistic explanation for the protective role of ApoE2 and the risk associated with ApoE4. These findings underline sialic acid as a crucial posttranslational determinant of ApoE function, influencing Aβ aggregation and possibly microglial SIGLEC-mediated responses [60] (Figure 2). At the synaptic level, the neural cell adhesion molecule (NCAM) and its polySia modification were found to be essential for synaptic plasticity. Loss of polySia in the medial prefrontal cortex impaired long-term potentiation through GluN2B-NMDAR overactivation, whereas application of short soluble polySia fragments restored normal synaptic signaling and cognitive performance in Alzheimer’s disease mouse models, suggesting therapeutic potential for polySia supplementation [61]. 

Altogether, these findings demonstrate that sialic acid and polySia plays multifaceted roles in the pathophysiology and diagnosis of Alzheimer’s disease. Aberrant sialylation affects not only classical neuropathological features such as tau tangles and amyloid plaques but also influences microglial activation, systemic inflammation, and synaptic function. Collectively, these findings highlight that sialic acid is not merely a structural sugar moiety but an active participant in neurodegenerative mechanisms and a promising diagnostic target. 

### 3.2. Others Neurodegenerative Diseases

Beyond Alzheimer’s disease, recent research has also revealed the involvement of sialic acid-related processes in other neurodegenerative disorders, particularly, Parkinson’s disease. Although the majority of studies have focused on amyloidogenic and tau-related pathologies, growing evidence highlights that disturbances in glycosylation and sialylation may also contribute to dopaminergic neurodegeneration. In one study, transcriptomic profiling of Parkinson’s disease brains revealed widespread alterations in the expression of glycobiology-related genes involved in glycosylation, sialylation, and sphingolipid metabolism. Specifically, decreased expression of polysialyltransferases (ST8SIA2, ST8SIA4) and the sialidase NEU4 was observed in the substantia nigra and putamen, alongside dysregulation of glycosyltransferases (B3GALT2, B4GALT1) and sphingosine pathway enzymes (SPHK1, SPHK2, SGPL1). These abnormalities suggest that impaired regulation of sialic acid metabolism and associated glycosylation processes may disturb neuronal membrane composition, lysosomal function, and inflammatory signaling [18].

### 3.3. Psychiatric Disorders

In addition to neurodegenerative conditions, dysregulation of sialic acid and its polymeric derivatives has been linked to many psychiatric disorders, including schizophrenia, bipolar disorder, ASD, and stress-related pathologies. Among these, polySia—a linear homopolymer of α-2,8-linked sialic acid residues predominantly attached to the neural cell adhesion molecule (NCAM)—emerges as a crucial modulator of neuroplasticity, cell migration, and synaptic remodeling. In the healthy adult brain, polySia-NCAM is retained in regions of ongoing neurogenesis such as the hippocampus, prefrontal cortex, and amygdala, supporting neural circuit flexibility and emotional regulation. Dysregulation of this system has been consistently observed in schizophrenia and related psychiatric conditions. In multiple studies, patients with schizophrenia exhibited altered serum and brain levels of polySia-NCAM, independent of pharmacological treatment, which correlated with negative symptoms, cognitive deficits, and reduced prefrontal cortical volume [62,63]. Genome-wide analyses identified variants in polysialyltransferase genes (e.g., ST8SIA2, ST8SIA3, ST8SIA4) as risk factors for schizophrenia and bipolar disorder [64,65,66]. These enzymes catalyze NCAM polysialylation, and their dysregulation leads to abnormal synaptic plasticity and impaired signaling of neuromodulators such as dopamine, brain-derived neurotrophic factor (BDNF), and fibroblast growth factor 2 (FGF2), all of which are central to psychiatric symptomatology. Notably, aberrant expression of polySia and its synthetic enzymes has been linked to structural and functional changes in key limbic regions—including the hippocampus and amygdala—across schizophrenia, bipolar disorder, and major depressive disorder, indicating partially overlapping molecular mechanisms underlying distinct psychiatric phenotypes [63,65,67]. Metabolomic studies further support this biochemical link: decreased levels of free N-acetylneuraminic acid in cerebrospinal fluid were detected in patients with early-phase psychosis, suggesting altered sialic acid metabolism at disease onset [68]. Advanced quantitative analyses also revealed age- and disease-dependent variations in brain polySia, with increased levels observed in schizophrenia models and human patients, potentially reflecting compensatory mechanisms in response to impaired neuroplasticity [69].

**Figure 3:**
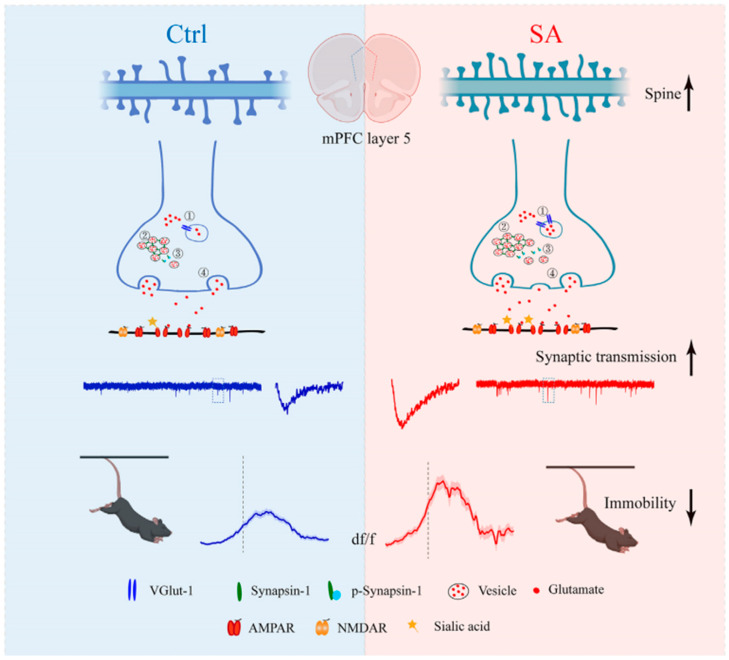
The schematic diagram of sialic acid enhanced the antistress capability under challenging situations by increasing synaptic transmission. Ctrl, control; mPFC, medial prefrontal cortex; SA, sialic acid; VGlut-1, vesicular glutamate transporter 1; AMPAR, α-amino-3-hydroxy-5-methyl-4-isoxazole-propionic acid receptor; NMDAR, N-methyl-D-aspartate receptor [71].

Similar mechanisms involving sialic acid metabolism have been observed in ASD, where altered sialylation and ganglioside-associated immune responses appear to contribute to neurodevelopmental dysregulation. Children with ASD exhibit significantly elevated plasma levels of total sialic acid and anti-ganglioside M1 (anti-GM1) IgG antibodies compared with healthy controls, with both biomarkers correlating with the severity of autistic behaviors [17]. The presence of anti-GM1 antibodies—frequently associated with autoimmune neuropsychiatric disorders—suggests an interplay between immune activation and disrupted glycosylation in ASD. Moreover, sialic acid supplementation in valproic acid (VPA)-induced autism models was shown to ameliorate ASD-like behaviors, normalize hippocampal neuronal morphology, and regulate the expression of GNE and St8sia2 genes involved in sialic acid biosynthesis and polysialylation [70]. Emerging data also indicate a link between sialic acid and emotional regulation in stress-related conditions. Early-life supplementation with sialic acid was found to enhance resilience to stress in animal models, improving exploratory and coping behaviors in standardized anxiety tests. This effect was associated with enhanced excitatory synaptic transmission and increased expression of synaptic proteins in the medial prefrontal cortex—an area critical for emotional control and stress response. Although these results derive from experimental models, they suggest that sialic acid may promote synaptic plasticity and strengthen neural circuits underlying adaptive stress responses, pointing to its potential relevance in affective and anxiety disorders [71] (Figure 3). 

Taken together, the cumulative evidence from schizophrenia, bipolar disorder, ASD, and stress-related models supports the concept that dysregulation of sialic acid metabolism and polysialylation represents a convergent molecular mechanism across neuropsychiatric and neurodevelopmental conditions. Nevertheless, despite extensive evidence on the role of sialic acid and polySia in common neurodegenerative and psychiatric disorders–primarily Alzheimer’s disease—available data remain limited for other conditions. While some studies address Parkinson’s disease and major psychiatric disorders such as schizophrenia, bipolar disorder, and ASD, information on less prevalent types of dementia or other psychiatric conditions is scarce. This highlights a clear gap in our understanding of sialic acid as a potential biomarker beyond the most commonly studied disorders. Key pathophysiological mechanisms related to sialic acid and polySia abnormalities in neurodegenerative and psychiatric disorders are outlined in Table 1.

## 4. Sialic Acid-Based Nanotherapeutic and Glycoconjugate Strategies for Neurodegenerative Diseases

Sialic acid has recently gained considerable attention as both a therapeutic molecule and a targeting motif in the treatment of neurodegenerative and psychiatric disorders. Among emerging therapeutic strategies, nanotechnology has become a particularly powerful tool for enhancing the delivery, selectivity, and efficacy of neuroprotective agents. The development of nanoscale carriers capable of crossing the BBB represents one of the most transformative advances in modern neuropharmacology. Conventional pharmacotherapy for neurological and psychiatric disorders is often hindered by poor brain penetration, rapid systemic clearance, and off-target toxicity. Nanostructured systems—such as liposomes, polymeric nanoparticles, dendrimers, and metallic nanocarriers—can be engineered to overcome these limitations by improving pharmacokinetic profiles, prolonging circulation time, and achieving controlled release within specific brain regions. Functionalization of these nanocarriers with sialic acid further enhances their therapeutic potential, as sialic acid exhibits high affinity for sialic acid-recognizing receptors on neuronal and endothelial cells, including sialoadhesin, selectins, and SIGLEC family proteins. This selective interaction facilitates transcytosis across the BBB and promotes accumulation of the nanocarriers in neural tissues, where they can exert site-specific therapeutic effects. Moreover, the negatively charged carboxyl group of sialic acid contributes to colloidal stability and biocompatibility, reducing unwanted aggregation and immune clearance.

### 4.1. Inorganic Nanocarriers

Yin et al. investigated the potential of sialic acid-modified selenium nanoparticles coated with a B6 peptide (B6-SA-SeNPs) as a therapeutic platform for Alzheimer’s disease. Selenium nanoparticles (SeNPs) were chosen due to their low toxicity, antioxidative properties, and ability to modulate redox balance in neuronal cells. The authors synthesized B6-SA-SeNPs as a synthetic selenoprotein analogue, with the B6 peptide enhancing BBB permeability. *In vitro* experiments demonstrated high cellular uptake of B6-SA-SeNPs by cerebral endothelial cells (bEnd.3) and efficient transport across a Transwell BBB model, followed by internalization in PC12 neuronal cells. Furthermore, functional assays revealed that B6-SA-SeNPs effectively inhibited amyloid-β (Aβ) aggregation and could disaggregate preformed Aβ fibrils into non-toxic amorphous oligomers. Additionally, treatment with B6-SA-SeNPs protected PC12 cells from Aβ-induced cytotoxicity. These results suggest that sialic acid functionalization, combined with the B6 peptide, not only facilitates nanoparticle transport across the BBB but also contributes to neuroprotection by reducing oxidative stress and Aβ toxicity. Overall, the study highlights B6-SA-SeNPs as a promising platform for targeted drug delivery and disease modification in Alzheimer’s disease [72]. 

### 4.2. Lipid-Based Nanocarriers

Similarly, in another study, authors developed sialic acid-modified asiatic acid nanostructured lipid carriers to mitigate cognitive deficits in Alzheimer’s disease. Asiatic acid, a pentacyclic terpenoid, is known for its antioxidant, anti-inflammatory, and anti-acetylcholinesterase activities, as well as regulation of tau phosphorylation, but its therapeutic potential is limited by poor solubility and rapid metabolism. To improve brain delivery, asiatic acid was first incorporated into cationic nanostructured lipid carriers using a hot-melt emulsification method, followed by surface conjugation with sialic acid via a carbodiimide-mediated reaction. *In vitro* studies using SH-SY5Y neuronal cells and hCMEC/D3 human brain microvascular endothelial cells demonstrated enhanced cellular uptake and BBB permeability of sialic acid-decorated nanostructured lipid carriers compared to non-sialylated formulations. Moreover, neuroprotective assessments revealed that sialic acid-modified asiatic acid nanostructured lipid carriers effectively reversed Aβ-induced cytotoxicity, improved cholinergic function by modulating acetylcholinesterase and butyrylcholinesterase levels, and reduced neuroinflammatory markers such as interleukin-6 and tumor necrosis factor-α. *In vivo* pharmacodynamic studies in Aβ-injected rats confirmed that sialic acid-functionalized nanostructured lipid carriers significantly improved cognitive performance and reduced features of Alzheimer’s disease, including Aβ, tau protein, and glycogen synthase kinase-3β levels [73]. Furthermore, sialic acid functionalization has been applied to enhance brain-targeted delivery of flavonoid-based therapeutics in Alzheimer’s disease models. In this study, cationic nanostructured lipid carriers (Cat-MY-NLC) encapsulating the antioxidant myricetin (MY) were surface-modified with sialic acid to yield Sia-Cat-MY-NLC. The sialic acid conjugation increased drug accumulation and residence time in plasma and brain. *In vitro* studies using SH-SY5Y cells demonstrated enhanced cellular uptake and neuroprotective effects, while transendothelial electrical resistance assays with hCMEC/D3 cells confirmed improved BBB permeability. *In vivo*, Sia-Cat-MY-NLC administration in Aβ1–42-induced Alzheimer’s disease rats resulted in higher bioavailability, enhanced brain targeting, and significant improvement in cognitive parameters, accompanied by restoration of Alzheimer’s disease-related biochemical markers [74]. In another study, sialic acid was utilized to engineer multifunctional nanocarriers combining photothermal and photodynamic therapy for Alzheimer’s disease. Sialic acid-modified polydopamine nanoparticles co-loaded with methylene blue and berberine (SA-BP-MB/BBR NPs) were designed to simultaneously inhibit Aβ aggregation, depolymerize fibrils, and suppress tau hyperphosphorylation under near-infrared (NIR) irradiation. The sialic acid coating enhanced BBB permeability and brain accumulation, while the photothermal effect of polydopamine transiently increased BBB permeability, enabling improved drug delivery. *In vivo* studies confirmed that SA-BP-MB/BBR NPs prolonged systemic circulation, increased brain uptake, and effectively mitigated Alzheimer’s disease pathology, underscoring their potential as a synergistic, multi-target therapeutic platform [75]. 

### 4.3. Dendrimeric Nanocarriers

**Figure 4:**
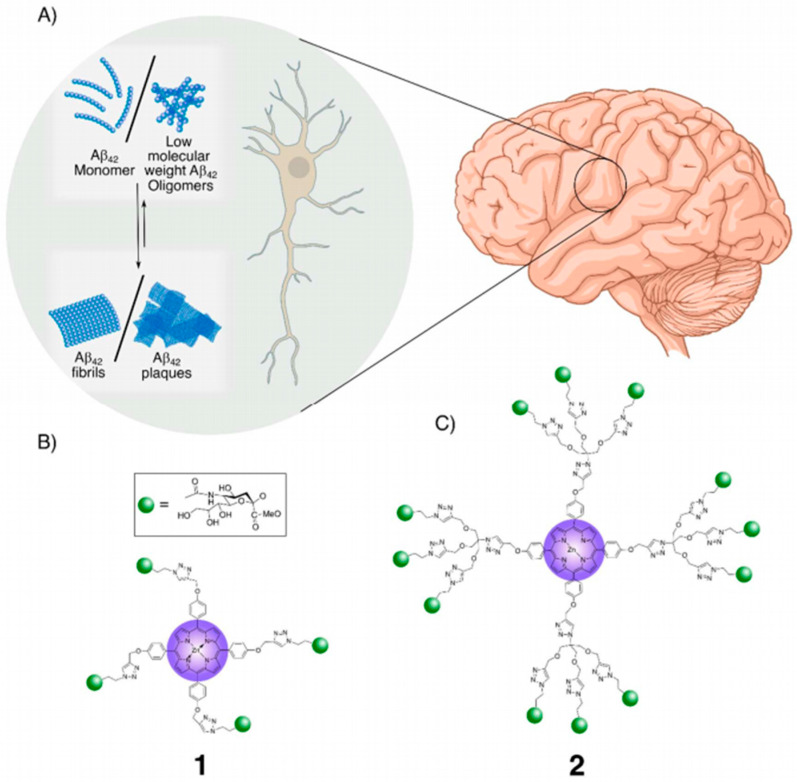
Porphyrin-cored sialic acid dendrimers 1 and 2 for the photo-degradation of Aβ42 plaques: (**A**). Schematic representation of Aβ42 plaque formation pathway in the extracellular space of neurons. Sialic acid moieties enable specific binding of 1 and 2 to Aβ42 plaques/fibrils, while the porphyrin ring core facilitates the absorption of UV irradiation, generating Reactive Oxygen Species (ROS) that promote the degradation of fibrils/plaques. (**B**). The glycoporphyrin dendrimers with sialic acid appendages: First-generation dendrimer, 1, and (**C**). second-generation dendrimer [76].

Sialic acid-functionalized glycodendrimers have been explored as light-activated therapeutic agents targeting Aβ42 fibrils in Alzheimer’s disease. First- and second-generation porphyrin-cored dendrimers carrying multiple sialic acid moieties were synthesized via copper(I)-catalyzed “click” chemistry. The peripheral sialic acid facilitated high-affinity binding to Aβ42 fibrils through the glycoside cluster effect, positioning the photoreactive porphyrin core in proximity to the fibrils. Upon irradiation, the porphyrin core generated singlet oxygen species, which disrupted hydrogen bonds and oxidized the peptide backbone, leading to the photolytic degradation of β-sheet-rich fibrils into lower-order soluble oligomers. High-resolution imaging, particle size analysis, and secondary structure studies confirmed the fragmentation of Aβ42 fibrils. Importantly, the glycodendrimers were non-toxic, even upon irradiation, and rescued SH-SY5Y neuroblastoma cells from Aβ42-induced cytotoxicity [76] (Figure 4).

### 4.4. Polymeric Nanoparticles and Polysaccharide-Based Systems

In a related approach, sialic acid has been employed to enhance brain-targeted delivery of antioxidant-loaded nanoparticles in Alzheimer’s disease models. In this study, poly(lactide-co-glycolide) (PLGA) nanoparticles (NPs) incorporating phosphatidic acid (PA) were surface-modified with sialic acid and 5-hydroxytryptamine-moduline (5HTM) to improve the delivery and activity of quercetin (QU) against oxidative stress induced by amyloid-β (Aβ) deposits. The resulting QU-SA-5HTM-PA-PLGA NPs exhibited uniform spherical morphology and enhanced aggregation due to PA incorporation. Importantly, surface conjugation with SA facilitated receptor-mediated endocytosis across the BBB via sialoadhesins on endothelial cells, while 5HTM enhanced recognition of brain microvascular endothelial cells through 5-HT1B receptor interactions. In Alzheimer’s disease rat models, QU-SA-5HTM-PA-PLGA NPs demonstrated efficient BBB penetration, reduced Aβ plaque formation, attenuated acetylcholinesterase and malondialdehyde activity, and suppressed caspase-3 expression, collectively providing neuroprotection [77]. 

Another approach has examined polySia as a biologically active polysaccharide with neuroprotective and immunomodulatory properties. In Parkinson’s disease, low molecular weight polySia (polySia avDP20) was investigated for its neuroprotective effects in humanized SIGLEC11 transgenic mice. PolySia, a linear polymer of α2,8-linked N-acetylneuraminic acid, naturally attaches to glycoproteins on neurons and immune cells and plays key roles in synaptic plasticity and inhibition of innate immunity. The authors demonstrated that polySia avDP20 interacts with the ITIM-bearing SIGLEC11 receptor on microglia, preventing inflammation, phagocytosis, and oxidative burst *in vitro*. *In vitro*, polySia avDP20 reduced lipopolysaccharide-induced Tnf mRNA expression in murine microglial cell lines and prevented neurite loss in macrophage-neuron co-cultures. *In vivo*, repeated systemic injections of lipopolysaccharide induced sustained brain inflammation in both wild type and SIGLEC11 transgenic mice, with elevated transcription of immune-related and cell death-related genes. Intraperitoneal administration of 10 μg/g body weight polySia avDP20 significantly attenuated this inflammatory response by day 19, reduced microglial activation (as shown by IBA1 and CD68 immunoreactivity), and prevented dopaminergic neuron loss in the substantia nigra pars compacta. These findings suggest that polySia avDP20 exerts neuroprotective effects through SIGLEC11-mediated immunomodulation and complements inhibition, highlighting its potential as a therapeutic candidate to mitigate inflammation-driven neurodegeneration in Parkinson’s disease [78]. Building on these insights into the biological activity of polySia, recent studies have focused on synthetic polySia-based glycoconjugates that combine the favorable properties of polySia with improved structural stability and therapeutic specificity. PolySia has also been utilized in the design of functionalized glycoconjugates to inhibit amyloid-β (Aβ) aggregation and attenuate neuroinflammation in Alzheimer’s disease. In this approach, polySia was grafted with mannuronate oligosaccharides (MOSs) to generate a negatively charged PSA-MOS conjugate, combining the biocompatibility of PSA with the structural stability of MOS. PolySia-MOS effectively impeded the α-helix to β-sheet transition of Aβ42 monomers. *In vitro* studies using SH-SY5Y cells demonstrated that polySia-MOS was non-cytotoxic and promoted cell proliferation. Moreover, in Aβ42-induced neurotoxicity models, polySia-MOS dose-dependently reduced cytotoxicity and significantly decreased levels of proinflammatory cytokines, including IL-1β, IL-6, and TNF-α. These effects were mediated by electrostatic interactions and hydrogen bonding between PSA-MOS and the N-terminal and central regions of Aβ42, which hindered intra-peptide β-sheet formation. Additionally, polySia-MOS exhibited metal-chelating capabilities, forming complexes with divalent cations such as Cu^2+^, further contributing to its anti-aggregation properties [79]. In another strategy aimed at mitigating amyloid-induced neurotoxicity, MOS conjugated with sialic acid (MOS-Sia) was investigated for its ability to inhibit Aβ42 aggregation and attenuate microglial inflammation. MOS, a polysaccharide derived from seaweed, possesses inherent antioxidant and anti-inflammatory properties. In this study, terminal activation of MOS enabled the synthesis of three MOS-Sia graft conjugates, of which MOS-Sia3 exhibited the most pronounced activity. The authors demonstrated that MOS-Sia3 effectively reduced β-sheet formation in Aβ42, inhibited oligomerization, and improved complexation with Ca^2+^ ions in solution, which are implicated in amyloid plaque pathology. *In vitro* experiments using BV-2 microglial cells showed that lower concentrations of MOS-Sia3 (25–50 µg/mL) significantly suppressed microglial activation induced by Aβ42, as evidenced by reductions in proinflammatory cytokines IL-1β, IL-6, and TNF-α. Higher concentrations (>50 µg/mL) additionally inhibited microglial proliferation without inducing overt cytotoxicity. These findings indicate that MOS-Sia3 not only directly interferes with amyloid fibrillation but also modulates microglial inflammatory responses, highlighting its potential as a multifunctional therapeutic candidate for the treatment of Alzheimer’s disease [80]. MOS have also been functionalized with sialic acid to develop conjugates capable of inhibiting Aβ42 aggregation. Low molecular weight mannan oligosaccharides (LBOSs) with a degree of polymerization of 3–13 were obtained via stepwise enzymatic hydrolysis of locust bean gum. LBOS was subsequently activated and chemically conjugated with sialic acid through fluoro–mercapto coupling, followed by phosphorylation to yield phosphorylated LBOS-SA (pLBOS-SA). Both LBOS-Sia and pLBOS-Sia demonstrated the ability to inhibit Aβ42 aggregation. In BV-2 microglial cells, these conjugates were non-cytotoxic and effectively reduced the release of the proinflammatory cytokine TNF-α induced by Aβ42, indicating anti-neuroinflammatory activity. Notably, pLBOS-SA inhibited the β-sheet transition of Aβ42 and promoted a shift toward α-helical or irregular conformations, highlighting its potential to interfere with pathological Aβ aggregation [81]. Previous studies also explored polySia as an endogenous carrier as an endogenous and biocompatible nanocarrier platform for the treatment of vascular dementia. In this study, polySia was chemically conjugated with octadecylamine to form an amphiphilic PSA-ODA copolymer capable of self-assembling into micelles for brain-targeted drug delivery. The resulting PSA–ODA micelles efficiently encapsulated the calmodulin antagonist DY-9836, exhibited sustained drug release, and demonstrated active BBB penetration via endocytosis and transcytosis. In a vascular dementia mouse model, treatment with PSA-ODA/DY micelles significantly improved spatial learning, restored hippocampal CaMKII and synapsin I phosphorylation, and increased neurogenesis, collectively indicating potent neuroprotective and cognitive benefits. These findings suggest that PSA-based micelles hold strong potential for brain-directed therapy in vascular dementia and other neurodegenerative conditions [82]. 

Collectively, these studies demonstrate the versatile applications of sialic acid in neurodegenerative disease therapeutics, including nanocarrier-mediated delivery, glycoconjugate-based inhibition of amyloid aggregation, and modulation of microglial receptors. Sialic acid-functionalization enhances BBB penetration, neuroprotective activity, and anti-inflammatory effects, while polySia and glycodendrimers offer additional strategies to directly interfere with Aβ aggregation or promote targeted photodegradation. These advances underscore that while current research has predominantly focused on Alzheimer’s disease, the promising outcomes of sialic acid-based strategies could be extended in the future to other neurodegenerative disorders, such as Parkinson’s disease, and potentially to psychiatric conditions. The summary of recent advances employing sialic acid–functionalized nanocarriers and glycoconjugate systems for neurodegenerative diseases is provided in Table 2.

## 5. Limitations, Challenges, and Future Perspectives of Sialic Acid-Based Strategies in Neurodegenerative and Psychiatric Disorders

Despite the remarkable progress achieved in understanding the multifaceted role of sialic acid and polysialylation in the pathophysiology and treatment of neurodegenerative and psychiatric disorders, several key challenges limit their translation from experimental to clinical conditions. Current knowledge largely derives from *in vitro* and *in vivo* studies employing immortalized murine neuronal or microglial cell lines (e.g., BV-2, PC12) or transgenic mouse models [72,80]. While these models provide valuable mechanistic insights, they cannot fully recapitulate the complexity of human neurobiology, particularly in terms of species-specific differences in sialylation patterns, glycosyltransferase expression, and receptor distribution [83,84]. The human SIGLEC receptor repertoire, for instance, differs significantly from that of rodents—many human SIGLECs have no functional murine orthologs. Consequently, therapeutic strategies targeting sialic acid-recognizing receptors may exhibit divergent outcomes in human systems compared to preclinical models. Similarly, sialic acid metabolism and polysialylation dynamics are tightly regulated by environmental and developmental cues that are difficult to model *ex vivo*, limiting the predictive value of current assays (Table 1). The available evidence regarding sialic acid-based therapeutic or diagnostic strategies derives exclusively from *in vitro* and *in vivo* preclinical studies, and no clinical trials have been conducted to date. This lack of clinical translation reflects several key barriers, including species-specific differences in sialylation patterns and SIGLEC receptor repertoires, insufficiently human-relevant BBB models, and limited long-term pharmacokinetic, toxicological, and immunological characterization of sialic acid-modified nanomaterials. Consequently, while preclinical findings are promising, their relevance to human neurobiology and therapeutic efficacy remains uncertain, underscoring the need for advanced humanized models and rigorous translational frameworks before clinical evaluation can be initiated. 

Another important limitation relates to the use of nanocarriers. From a therapeutic standpoint, most sialic acid-based strategies developed so far have focused on antioxidative and anti-amyloid mechanisms or modulation of SIGLEC-mediated microglial responses (Table 2). However, the broader potential of sialic acid in regulating neuroplasticity, synaptogenesis, and neuroimmune crosstalk remains largely unexplored. Despite growing evidence linking aberrant polysialylation with psychiatric disorders such as schizophrenia, depression, and autism spectrum disorders, few studies have investigated the therapeutic restoration of polysialylation or targeted modulation of polysialyltransferases *in vivo*. Similarly, the majority of nanotechnology-driven approaches have yet to integrate the complex signaling roles of sialic acid within neuronal networks, focusing instead on its use as a passive targeting ligand. It should also be noted that nanocarriers, in general, have additional inherent limitations, including insufficient specificity to target cells without appropriate ligands, challenges in large-scale production with consistent particle size, morphology, and surface properties, limited integration into physiological signaling pathways, and a lack of comprehensive regulatory and safety data required for clinical translation. This highlights the need for next-generation glycoconjugate and nanocarrier systems capable of dynamic, stimuli-responsive behavior that mimics physiological sialylation cycles. Future research should therefore prioritize the development of human-relevant models, including induced pluripotent stem cell (iPSC)-derived neuronal and glial cultures, humanized mouse lines expressing human SIGLEC receptors, and advanced microfluidic BBB-on-chip platforms. These systems will enable more accurate assessment of sialic acid-mediated transport and receptor interactions under physiologically relevant conditions. Addressing formulation challenges such as precise control of sialic acid density, stability, and linkage configuration will also be essential for improving reproducibility and translational viability. Finally, integrating sialic acid-based nanotherapeutics with emerging modalities—such as gene therapy or targeted degradation of pathogenic proteins—may unlock synergistic opportunities for restoring glycohomeostasis and neuroimmune balance in both neurodegenerative and psychiatric diseases. Collectively, while sialic acid-based therapeutic and diagnostic platforms represent a rapidly evolving and highly promising field, their clinical translation requires overcoming significant experimental and biological barriers. The next stage of research must therefore move beyond proof-of-concept nanocarriers toward mechanistically driven, clinically relevant interventions that leverage the full biological complexity of sialic acid in the human brain.

## 6. Conclusions

Previous reports clearly indicate the key role of sialic acid in the pathophysiology and potential treatment of neurodegenerative and psychiatric disorders. Terminal sialic acid residues on glycoproteins and glycolipids modulate neuronal signaling, synaptic plasticity, immune responses, and neuroinflammation, influencing both disease onset and progression. Alterations in sialylation patterns, whether in the form of aberrant glycosylation, dysregulated SIGLEC receptor signaling, or impaired polySia-mediated synaptic modulation, have been consistently associated with Alzheimer’s disease, Parkinson’s disease, schizophrenia, bipolar disorder, and autism spectrum disorder. Classical pharmacotherapies remain largely symptomatic, addressing neurotransmitter imbalances but not fully targeting the underlying cellular and molecular dysregulations. In this context, sialic acid-based strategies—particularly nanocarrier-mediated delivery systems, glycoconjugates, and polySia-based therapeutics—offer a promising avenue to go beyond symptomatic management. Preclinical studies have demonstrated enhanced BBB penetration, antioxidative effects, microglial modulation, and inhibition of amyloid aggregation. Nevertheless, most data come from mouse or *in vitro* models, with limited translation to human physiology, emphasizing the need for humanized systems and advanced translational models. Looking forward, integrating glycomics, proteomics, and high-resolution imaging with nanotechnology and rationally designed glycoconjugates may unlock the full potential of sialic acid in targeted and controlled therapeutic strategies. Such approaches could not only mitigate neurodegenerative and psychiatric pathology but also promote synaptic resilience and prevent disease progression. Overall, while the clinical application of sialic acid-based interventions is still in its infancy, ongoing research points to a future in which modulation of sialylation could become a cornerstone of precision therapy in neurodegenerative and psychiatric disorders.

## Figures and Tables

**Table 1:** Sialic acid–related mechanisms across neurodegenerative and psychiatric disorders.

Disorder	Mechanism	Effect	Notes	Reference
Alzheimer’s disease	Increased expression of SIGLEC10 and SIGLEC11	Anti-inflammatory signaling, reduced microglial activation, attenuated neuronal loss	SIGLEC10/11-mediated recognition may protect against neuroinflammatory damage	[45,46,47]
Alzheimer’s disease	SIGLEC3 (CD33) isoforms: SIGLEC3m, SIGLEC3M, SIGLEC3 R69G	The longer isoform inhibits phagocytosis of Aβ aggregates and enhances cell adhesion, while the shorter isoform promotes phagocytosis and proliferation. The risk variant reduces Aβ clearance.	Aberrant SIGLEC signaling contributes to impaired debris clearance and disease progression	[48,49,50,51]
Alzheimer’s disease	Aberrant sialylation changes (N- and O-linked), phosphorylated tau, microglial keratan sulfate (KS)	Hypersialylation in tau structures; KS suppresses microglial phagocytosis; regional variation in sialic acid	Sialylation patterns may serve as histological or functional indicators of disease progression	[52,53,54,55,56,57]
Alzheimer’s disease	Sialic acid metabolism, oxidative stress, ApoE sialylation, NCAM-polySia	Correlates with oxidative imbalance, modulates Aβ aggregation, influences synaptic plasticity	PolySia supplementation restores cognitive function in models; sialic acid is a crucial post-translational determinant influencing Aβ aggregation and possibly microglial SIGLEC-mediated responses	[58,59,60,61]
Parkinson’s disease	Polysialyltransferases (ST8SIA2, ST8SIA4), sialidase NEU4, glycosyltransferases, sphingosine pathway enzymes	Dysregulation alters membrane composition, lysosomal function, and inflammatory signaling	Impaired sialic acid metabolism contributes to dopaminergic neurodegeneration	[18]
Schizophrenia	PolySia-NCAM	Correlates with negative symptoms, cognitive deficits, reduced prefrontal cortical volume	Effect independent of pharmacological treatment	[62,63]
schizophrenia, bipolar disorder	Polysialyltransferase genes (ST8SIA2, ST8SIA3, ST8SIA4)	Abnormal NCAM polysialylation–impaired synaptic plasticity and neuromodulator signaling	Genetic risk factors; affects neuroplasticity and signaling	[64,65,66]
Schizophrenia, bipolar disorder, major depressive disorder	PolySia and synthetic enzymes in limbic regions (hippocampus, amygdala)	Structural and functional alterations in limbic regions	Overlapping molecular mechanisms across psychiatric disorders	[67]

**Table 2:** Sialic acid-based nanotherapeutic and glycoconjugate strategies for neurodegenerative diseases.

Nanocarrier and Glycoconjugate	Therapeutic Aim	Experimental Model	Key Findings	References
B6-SA-SeNPs (selenium nanoparticles with B6 peptide and sialic acid)	Inhibition of Aβ aggregation, reduction of oxidative stress	bEnd.3 and PC12 cells; *in vitro* BBB	High cellular uptake, BBB transport, disaggregation of preformed Aβ fibrils, protection of PC12 cells	[72]
Sialic acid-modified asiatic acid nanostructured lipid carriers	Improvement of cholinergic function, anti-neuroinflammatory effects, reduction of Aβ and tau	SH-SY5Y, hCMEC/D3; Aβ-injected rats	Enhanced cellular uptake and BBB penetration, improved cognitive function, reduced AD markers	[73]
Sia-Cat-MY-NLC (myricetin with sialic acid; cationic nanostructured lipid carriers)	Neuroprotection, increased bioavailability and brain accumulation	SH-SY5Y, hCMEC/D3; Aβ1–42 rats	Enhanced BBB penetration, improved cognitive parameters, restoration of AD-related biomarkers	[74]
SA-BP-MB/BBR NPs (sialic acid-modified polydopamine nanoparticles co-loaded with methylene blue and berberine)	Inhibition of Aβ aggregation, fibril depolymerization, reduction of tau hyperphosphorylation	AD rats	Increased brain accumulation, mitigation of AD pathology	[75]
Glycodendrimers (porphyrin-coated with sialic acid)	Light-activated degradation of Aβ42 fibrils	SH-SY5Y; *in vitro*	Fragmentation of Aβ fibrils, non-toxic, rescued cells from Aβ-induced cytotoxicity	[76]
QU-SA-5HTM-PA-PLGA NPs (quercetin with sialic acid and 5HT-moduline)	Reduction of oxidative stress, neuroprotection	AD rat models	BBB penetration, reduced Aβ plaques, decreased acetylcholinesterase activity, neuroprotection	[77]
polySia-MOS (polySia grafted with mannuronate oligosaccharides)	Inhibition of Aβ aggregation, modulation of microglia	SH-SY5Y; *in vitro*	Reduced cytotoxicity, decreased IL-1β, IL-6, TNF-α, metal chelation	[79]
MOS-Sia3 (MOS conjugated with sialic acid)	Inhibition of β-sheet formation in Aβ42, reduction of microglial activation	BV-2; *in vitro*	Suppressed proinflammatory cytokines, inhibited microglial proliferation	[80]
LBOS-Sia/pLBOS-SA (oligomannuronic acid–sialic acid conjugate)	Inhibition of Aβ42 aggregation, reduction of neuroinflammation	BV-2; *in vitro*	Blocked β-sheet transition, reduced TNF-α release	[81]
PSA–ODA micelles (polySia conjugated with octadecylamine)	Delivery of DY-9836, cognitive protection	Vascular dementia mouse model	Improved spatial learning, increased neurogenesis, active BBB penetration	[82]

## Data Availability

No new data were created or analyzed in this study. Data sharing is not applicable to this article.
